# Retrospective study comparing rectal endoscopic submucosal dissection with and without Foley catheter drainage tube placement

**DOI:** 10.1055/a-2631-7694

**Published:** 2025-07-01

**Authors:** Yuka Kagaya, Hiroaki Ishii, Yoshikazu Hayashi, Hiroki Hayashi, Satoshi Sato, Stefano Kayali, Kohei Suzuki, Takaaki Morikawa, Masahiro Okada, Takahito Takezawa, Ayman Qawasmi, Keijiro Sunada, Hirotsugu Sakamoto, Tomonori Yano, Hironori Yamamoto

**Affiliations:** 1 215677 Department of Medicine, Division of Gastroenterology, Jichi Medical University Hospital, Shimotsuke, Japan; 2 12838 Department of Medicine, Division of Gastroenterology, Jichi Medical University, Shimotsuke, Japan; 3 9370 Department of Medicine and Surgery, University of Parma, Parma, Italy; 4 38219 Coloproctology, Fukushima Medical University Aizu Medical Center, Aizuwakamatsu, Japan; 5 Gastroenterology, Samson Assuta Ashdod University Hospital, Ashdod, Israel; 6 12838 Gastroenterology, Jichi Medical University, Shimotsuke, Japan; 7 4. Masuyama Gastrointestinal Clinic, Masuyama Gastrointestinal Clinic, Ohtawara, Japan; 8 12838 Department of Endoscopic Research and International Education Funded by FUJIFILM Medical Co., Ltd., Jichi Medical University, Shimotsuke, Japan

**Keywords:** Endoscopy Lower GI Tract, Polyps / adenomas / ..., Colorectal cancer, Endoscopic resection (polypectomy, ESD, EMRc, ...)

## Abstract

**Background and study aims:**

Endoscopic submucosal dissection (ESD) is a minimally invasive and effective treatment for rectal tumors but maintaining a clear surgical field during the procedure is challenging, especially for novice operators. This study aimed to investigate whether continuous drainage using a Foley catheter could enhance efficiency and safety of rectal ESD performed by novice endoscopists under expert supervision.

**Patients and methods:**

This retrospective study involved 26 patients who underwent rectal ESD between March 2023 and October 2024. Patients were divided into two groups: those who received continuous drainage with Foley catheter placement (n = 12) and those who did not (n = 14). Key outcomes evaluated were dissection speed, total procedure time, R0 resection rates (complete tumor resection with clear margins), and occurrence of any adverse events (AEs) during or after the procedure. All procedures were performed by novice endoscopists under expert guidance.

**Results:**

The results showed that dissection speed was significantly higher in the Foley catheter group, with a median of 18.6 mm²/min compared with 10.5 mm²/min in the non-catheter group ( *P* = 0.027). Although total procedure time and sodium hyaluronate usage were lower in the catheter group, these differences were not statistically significant. Importantly, no AEs were reported in either group.

**Conclusions:**

Foley catheter placement notably improved efficiency of rectal ESD performed by novice endoscopists, particularly by increasing dissection speed. This technique may contribute to safer and more effective ESD. However, larger studies are needed to confirm these findings and further assess their benefits.

## Introduction

Endoscopic submucosal dissection (ESD) has become a standard, minimally invasive treatment for superficial colorectal cancer worldwide 1 2 . Procedure success relies heavily on maintaining stable endoscopic maneuverability and a clear surgical field, which can be challenged by presence of blood and other fluids. Although various ESD strategies exist, some can be facilitated by collapsing the intestinal lumen and/or removing blood 3 . Recent advancements suggest that saline immersion may significantly improve safety and efficiency of ESD procedures 4 5 . However, this technique necessitates meticulous control over the volume of irrigated saline and washing away of blood and gas bubbles generated by water electrolysis during the procedure. Accumulation of hydrogen and oxygen bubbles poses a risk of explosion 6 7 8 . In addition, relying solely on the endoscope accessory channel to aspirate gas, blood, and saline may obscure the view, disrupting the dissection process. To address these challenges, we developed a drainage-tube ESD technique for rectal tumors designed to spontaneously remove excess saline, including intestinal fluid, blood, and insufflated or generated gases 9 . The aim of this study was to retrospectively assess effectiveness and efficiency of rectal ESD performed by novice endoscopists, with and without use of a Foley catheter as a drainage tube.

## Patients and methods

### Patients

The records of all patients who underwent colorectal ESD at Jichi Medical University, Japan, between March 2023 and October 2024 were reviewed. The analysis included all patients who underwent a rectal ESD by novice endoscopists who had no prior experience performing colorectal ESD as of March 1, 2023. At our university hospital, standard rectal ESD procedures are assigned to novice endoscopists as part of their training program. The decision to use a Foley catheter for drainage was left to the discretion of each novice endoscopist, based on their individual interest in the technique and their prior observations of ESD procedures performed by other endoscopists. A total of 26 patients were included in the study. Written informed consent for rectal ESD was obtained from all patients.

### Operators and assistants

To standardize skill levels across the procedures, novice endoscopists with fewer than 20 gastric ESDs performed and no prior colorectal ESD experience were selected as operators. These endoscopists were not yet qualified to perform ESD independently. The participating ESD operators were independent endoscopists with experience in total colonoscopy, polypectomy, and endoscopic mucosal resection (EMR), and they were assisted by expert endoscopists who had performed more than 100 colorectal ESDs and had substantial experience supervising ESD procedures.

### ESD procedures

**Fig. 1:**
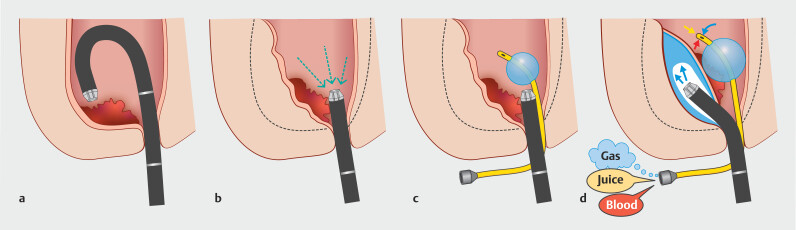
By continuously removing gas and fluid through a transanal Foley catheter during endoscopic submucosal dissection (ESD), the rectum can be collapsed. **a** Insufflation expands the rectal lumen, and lesions near the anal canal are often located behind the rectal valve, making the submucosa thinner and endoscopic manipulation more difficult. **b** Aspiration allows the rectal wall to align tangentially to the endoscope, resulting in a thicker submucosa and stable endoscopic control within the narrower space. **c,d** The Foley catheter, once inserted into the rectum, automatically drains gas and fluid, such as blood, without any additional interventions. This leads to a collapsed rectal lumen, improves stability in endoscopic maneuvering, thickens the submucosa, and prevents clot buildup. The irrigated saline displaces fluid, including blood, which is then drained via the catheter, thereby facilitating ESD. Because the catheter is non-conductive, it does not interfere with diathermy. Its safety has already been established because it is used as a urinary catheter. In the event of balloon rupture, the distilled water will simply leak into the rectum.

**Fig. 2:**
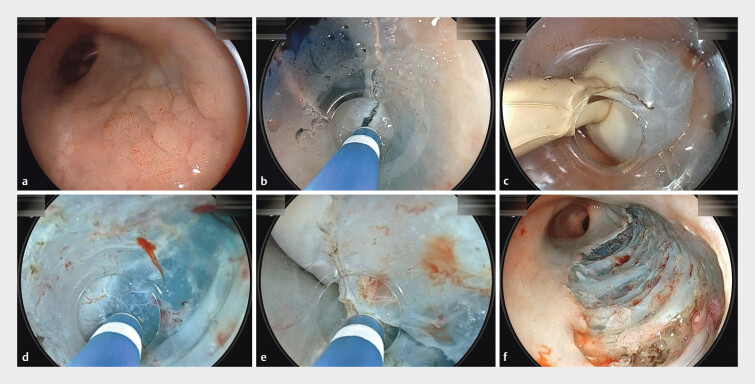
ESD of a laterally spreading tumor (LST) in the distal rectum. **a** Two-cm LST in the distal rectum. **b** Incision of the distal mucosa following submucosal injection of 0.4% sodium hyaluronate. **c** Placement of a Foley catheter in the rectum. **d** Submucosal dissection using the pocket-creation method, forming a submucosal pocket. **e** After pocket creation, a circumferential incision was made, and the pocket was subsequently opened, completing the ESD. **f** The post-ESD mucosal defect. Histology confirmed low-grade dysplasia with negative resection margins.

A gastroscope (EG-840T or EG-840TP; Fujifilm, Tokyo, Japan), a carbon dioxide insufflation regulation unit (GW-100; Fujifilm), and a mechanical water pump (JW-2; Fujifilm) with saline were used for all procedures. The VIO-3 (ERBE Elektromedizin GmbH, Tübingen, Germany) was employed for diathermy. The settings for the diathermy devices on the VIO-3 were as follows: mucosal incisions (endoCUT I, effect 1, duration 1–4, interval 1), submucosal dissection (swiftCOAG, 3.0–3.5), and coagulation (softCOAG, 3.5). Electrosurgical knives used included the DualKnife (KD-650Q; Olympus), FlushKnife (DK2620J-B15S; Fujifilm), ORISE ProKnife (M00519351; Boston Scientific, Marlborough, Massachusetts, United States), or single use electrosurgical knife known as “Gold Knife” (MK-I-1–195; Micro-Tech, Nanjing, China). RAICHO2 (RC2200–25C; KANEKA MEDIX CORP., Osaka, Japan) was used as hemostatic forceps. In both groups, a conical cap (ST hood, DH-33GR; Fujifilm) or CAST hood (TOP Corporation, Tokyo, Japan) and 0.4% sodium hyaluronate solution (MucoUp; Boston Scientific) for submucosal injection were used. For drainage-tube ESD, a general-purpose 16F Foley catheter was employed as a drain 10 . The catheter was inserted transanally, and the balloon was inflated from the start to the completion of the ESD procedure ( Fig. 1 , Fig. 2 ). An open plastic bag was used to collect drainage from the catheter. All the rectal ESDs were performed using saline-immersion technique 4 5 . For patient safety, the assistant (expert endoscopist) was permitted to take over from the operator in cases of intraoperative perforation, uncontrollable bleeding, or if the operator was unable to continue the procedure effectively. In addition, if procedure time exceeded 2 hours, the assistant could assume control to prevent operator fatigue and loss of concentration. These decisions were at the discretion of the assistant overseeing the procedure.

### Evaluation of ESD

The number of ESDs performed by each operator, with or without Foley catheter drainage tube placement, as well as the total amount of hyaluronic acid used in each ESD, were evaluated. Procedure time was measured from initiation of the first mucosal incision to completion of lesion excision. Dissection speed was calculated using the formula: = (short diameter of the excised specimen × long diameter of the excised specimen × 0.25 × 3.14) / procedure time (minutes), En bloc resection was defined as excision of the lesion in one single piece. R0 resection was described as histopathological confirmation of an en bloc resection with negative vertical and horizontal margins. Adverse events (AEs), such as delayed bleeding and perforation, were recorded. Delayed bleeding was defined as significant bleeding occurring within 14 days following ESD, resulting in a hemoglobin drop of at least 2 g/dL and necessitating either endoscopic hemostasis or a blood transfusion. Perforation was defined as a severe mucosal injury leading to direct communication between the intraperitoneal cavity and the intestinal lumen, either during or after the procedure; the former was identified as intraoperative perforation and the latter as delayed perforation. Intraoperative perforation was diagnosed when endoscopic observation confirmed presence of the intraperitoneal cavity. Delayed perforation was diagnosed based on detection of free air on a computed tomography scan or x-ray.

### Statistical analysis

The Mann-Whitney U-test was used to assess non-parametric data. The Fisher’s exact test was used to evaluate categorical data. Differences were considered significant when *P* < 0.05. All statistical analyses were performed with EZR (Saitama Medical Center, Jichi Medical University, Saitama, Japan), which is a graphical user interface for R (The R Foundation for Statistical Computing, Vienna, Austria). More precisely, it is a modified version of R commander designed to add statistical functions frequently used in biostatistics.

## Results

### Baseline patient characteristics

From March 2023 to October 2024, 26 patients with 26 rectal tumors were included in the analysis. Of these, 12 patients underwent ESD with Foley catheter placement, and 14 without. Baseline characteristics of patients, lesions, and the experience of each operator are shown in Table 1 . The cohort comprised 13 male and 13 female patients, with a mean age of 64 years (SD 12.5 years).

**Table:** **Table 1** Baseline characteristics.

	With Foley catheter n = 12	Without Foley catheter n = 14	*P* value
Age, median	60	64	0.877
Sex			
Male	6	7	1
Female	6	7	
Antithrombotic medication	2	1	0.58
Location			
Ra	3	2	0.635
Rb	9	12	
Morphology			
Flat	6	4	0.483
Elevated	4	5	
Submucosal tumor	2	5	

### Operators and devices

Seven endoscopists were included in this assessment. The colorectal/gastric ESD experience of each endoscopist was as follows: 0/10 (Y.K.), 0/18 (H.I), 0/10 (H.H.), 0/0 (S.K.), 0/3 (S.S.), 20/0 (K.S.), and 0/0 (A.Q.) respectively. Data on the operators and devices are presented in Table 2. Distribution of operators and types of knives used were not significantly different between the two groups ( *P* = 0.272 and *P* = 0.410).

### Therapeutic outcomes of patients

All ESDs were performed by the endoscopist with guidance from the assistants, without any takeover by them. ESD data are presented in Table 2 . Median (25%-75%) dissection speeds were 18 mm²/min (13.5–25.6) and 10.5 mm²/min (6.5–14.3) in the groups with and without Foley catheter placement, respectively, showing a significant difference ( *P* = 0.027). There were no significant differences in tumor size, specimen size, R0 resection rate, or histological results between the two groups. Although the differences were not statistically significant, the amount of sodium hyaluronic acid used and procedure time were smaller and shorter, respectively, in the Foley catheter group compared with the group without Foley catheter placement. No AEs were observed.

**Table:** **Table 2** ESD results.

	With Foley catheter n = 12	Without Foley catheter n = 14	*P* value
Operator			
A	2	1	0.272
B	5	3	
C	1	5	
D	0	1	
E	0	2	
F	3	2	
G	1	0	
Knife			
DualKnife	1	5	0.410
FlushKnife	1	2	
ProKnife	2	1	
Gold Knife	8	6	
Amount of hyaluronic acid used, median, mL	35.5	38.0	0.938
Procedure time, median, min.	45.5	65.0	0.607
Dissection speed, median, mm ^2^ /min.	18.6	10.5	0.005
En bloc resection	12	14	1
Adverse event	0	0	1
Tumor size, median, mm	24.5	22.0	0.291
Specimen size, median, mm	35.5	32.5	0.303
Histology			
Low-grade dysplasia	7	2	0.069
High-grade dysplasia	3	7	
Neuroendocrine tumor	2	5	
Lymphovascular invasion	0	1	1
R0 resection	12	13	1
Positive horizontal margin	0	1	1
ESD, endoscopic submucosal dissection.			

## Discussion

In this retrospective comparative study, we demonstrated that Foley catheter placement for continuous drainage significantly enhanced dissection speed during rectal ESD performed by novice operators. Although reductions in procedure time and hyaluronic acid use were not statistically significant, they indicate a trend toward improved efficiency. To our knowledge, this is the first comparative study to evaluate effectiveness of drainage-assisted ESD using Foley catheter placement.

Colorectal ESD has become a minimally invasive standard treatment 1 2 ; however, it is not without challenges, and in reality, treatment outcome of ESD is influenced by the skill level of the operator 10 . From the perspective of organ preservation, laparoscopic colectomy can be considered a treatment option comparable to ESD for early-stage colon cancer because it is not as dependent on operator skill as ESD 11 . However, for rectal lesions, curative ESD is essential to avoid total rectal resection, preserve anal function, and maintain patient quality of life 12 . Therefore, promoting reliable rectal ESD is a high priority. This background explains why this study was limited to rectal tumors and novice ESD operators.

In the evolution of colorectal ESD, strategic advancements such as the pocket-creation method 13 , water-pressure method 5 , and saline immersion therapeutic endoscopy 4 , as well as practical application of traction devices 14 15 , have made significant contributions in recent years. However, for more efficient ESD, it is desirable to have easy control over air insufflation volume in the pocket-creation method 3 and water infusion volume in saline immersion methods 4 5 .

In addition, regardless of the method used, it should be easy to remove generated gases and intraoperative bleeding. Masunaga, et al. demonstrated that in water-pressure method, use of a dedicated continuous liquid-suction catheter attachment effectively drains excess water and maintains clarity of the infused water 16 . We have shown that by placing a Foley catheter in the rectum, gases, water, and intraoperative bleeding can be efficiently evacuated during ESD 9 . Although dissection speed was significantly higher in the Foley catheter group, total procedure time did not differ significantly between groups. This discrepancy may be explained by several confounding factors, including differences in lesion characteristics, procedure complexity, and variation in endoscopist experience. In addition, procedures performed using techniques other than the pocket-creation method (PCM) may have inherently longer dissection times, which could offset time gained through improved dissection efficiency. Furthermore, use of the Foley catheter contributed to continuous rectal lumen collapse through gas and fluid drainage. This collapse facilitated a tangential alignment of the rectal wall to the endoscope, resulting in a thickened and more stable submucosal layer. Such conditions enhanced both maneuverability and dissection precision. To maintain an adequate field of view in the collapsed lumen, we routinely used a conical ST hood, which proved effective in securing a clear view of the submucosal layer throughout the procedure. The Foley catheter can help reduce need for active suctioning, benefiting not only novice endoscopists but also improving efficiency and ease of ESD procedures for experienced endoscopists.

However, the Foley catheter is not very effective for ESD procedures involving lesions close to the rectosigmoid junction due to the increased distance from the anus, where the Foley catheter becomes fixed. We have previously reported the effectiveness of the alpha-shaped tube as an alternative drainage method 17 . Because the tip of the alpha-shaped tube is positioned further from the anus than the Foley catheter, it is better suited for treating proximal rectal lesions. The balloon may shift due to endoscopic movement. However, drainage was generally not significantly impaired, because the range of to-and-fro motion of the endoscope during ESD was usually limited. Because the rectum is expandable like a large lumen, the catheter rarely interferes with procedures, even in the case of large rectal lesions. If the endoscopist finds the balloon bothersome, pushing the catheter shaft can displace the balloon proximally, away from the procedure area. In this study, catheter placement was applied only in cases performed by novice endoscopists. Based on these results, we recommend considering use of a Foley catheter primarily for trainees, particularly under the PCM and underwater conditions, because it may improve procedure stability and efficiency. In addition to facilitating gas evacuation, the catheter may assist in draining intrarectal water and blood from minor bleeding, helping to maintain a clear operative field. Although our findings are based on trainee cases, this technique may also be useful in selected expert-level procedures. Use of a Foley catheter did not significantly interfere with endoscopic maneuverability when standard lubrication was applied, because the range of endoscope movement during rectal ESD is generally limited. Although occasional slight obstruction of the visual field by the catheter was noted, it was minimal and did not affect the overall progress or safety of the procedure. All procedures were performed in the left lateral decubitus position, which may have helped maintain consistent endoscopic conditions across cases.

The Foley catheter used in this study costs approximately 2,000 Japanese yen per unit, which is not an expensive medical material in Japan. The α-shaped nasogastric tube we previously reported costs 88 Japanese yen 17 , which is even cheaper, but considering the effort required to customize the shape and uncertainty about maintaining that shape, the Foley catheter, a widely used medical material for bladder drainage in many institutions, seems to be more practical in many hospitals. Furthermore, compared with many ESD assist devices 15 18 , which are relatively expensive, the Foley catheter offers promising cost-effectiveness.

It is a well-known phenomenon in transurethral endoscopic procedures that saline solution introduced into the bladder can undergo electrolysis due to high-frequency electric current, producing hydrogen and oxygen, which may lead to a hydrogen explosion when ignited by sparks from an electrosurgical knife 6 7 . Nomura, et al. presented a case showing that a hydrogen explosion can occur during ESD for the same reason 8 . In gastrointestinal endoscopy, hydrogen explosions are likely a rare occurrence, because they would require several conditions to coincide, such as complete isolation of carbon dioxide gas (commonly used in fire extinguishers) and localized accumulation of high-purity hydrogen and oxygen. However, risk of hydrogen gas generation should be acknowledged. The Foley catheter provides a continuous pathway for gas evacuation, bypassing the anal sphincter, which may prevent hydrogen gas accumulation and reduce risk of intraprocedural explosion. The Foley catheter may also serve as an effective route for evacuation of hydrogen gas.

This study has several limitations. First, this was a retrospective, non-randomized study conducted at a single institution, in which all consecutive patients who underwent rectal ESD performed by novice endoscopists were included. Second, to avoid the learning-curve effect, where ESD skills improve with the increase in cases, we limited the study period to 20 months, which resulted in a small sample size of 26 cases. Third, to ensure patient safety, each novice rectal ESD operator completed the procedures under the guidance and supervision of an expert, rather than independently. Fourth, the type of ESD knife was not standardized, and both injectable and non-injectable knives were used. Fifth, the exact amount of saline irrigation and its recovery were not accurately recorded. Therefore, reliability of the results should be carefully considered, and further studies are warranted.

## Conclusions

According to this study, Foley catheter placement for continuous drainage appeared to improve efficiency of rectal ESD performed by novice operators by increasing dissection speed.
